# The Oldest, Slowest Rainforests in the World? Massive Biomass and Slow Carbon Dynamics of *Fitzroya cupressoides* Temperate Forests in Southern Chile

**DOI:** 10.1371/journal.pone.0137569

**Published:** 2015-09-09

**Authors:** Rocio Urrutia-Jalabert, Yadvinder Malhi, Antonio Lara

**Affiliations:** 1 Environmental Change Institute, School of Geography and the Environment, University of Oxford, Oxford, OX1 3QY, United Kingdom; 2 Laboratorio de Dendrocronología y Cambio Global, Instituto de Conservación, Biodiversidad y Territorio, Facultad de Ciencias Forestales y Recursos Naturales, Universidad Austral de Chile, Valdivia, Chile; 3 Center for Climate and Resilience Research (CR)^2^, Facultad de Ciencias Físicas y Matemáticas, Universidad de Chile, Santiago, Chile; 4 Fundación Centro de los Bosques Nativos FORECOS, Valdivia, Chile; University Copenhagen, DENMARK

## Abstract

Old-growth temperate rainforests are, per unit area, the largest and most long-lived stores of carbon in the terrestrial biosphere, but their carbon dynamics have rarely been described. The endangered *Fitzroya cupressoides* forests of southern South America include stands that are probably the oldest dense forest stands in the world, with long-lived trees and high standing biomass. We assess and compare aboveground biomass, and provide the first estimates of net primary productivity (NPP), carbon allocation and mean wood residence time in medium-age stands in the Alerce Costero National Park (AC) in the Coastal Range and in old-growth forests in the Alerce Andino National Park (AA) in the Andean Cordillera. Aboveground live biomass was 113–114 Mg C ha^-1^ and 448–517 Mg C ha^-1^ in AC and AA, respectively. Aboveground productivity was 3.35–3.36 Mg C ha^-1^ year^-1^ in AC and 2.22–2.54 Mg C ha^-1^ year^-1^ in AA, values generally lower than others reported for temperate wet forests worldwide, mainly due to the low woody growth of *Fitzroya*. NPP was 4.21–4.24 and 3.78–4.10 Mg C ha^-1^ year^-1^ in AC and AA, respectively. Estimated mean wood residence time was a minimum of 539–640 years for the whole forest in the Andes and 1368–1393 years for only *Fitzroya* in this site. Our biomass estimates for the Andes place these ecosystems among the most massive forests in the world. Differences in biomass production between sites seem mostly apparent as differences in allocation rather than productivity. Residence time estimates for *Fitzroya* are the highest reported for any species and carbon dynamics in these forests are the slowest reported for wet forests worldwide. Although primary productivity is low in *Fitzroya* forests, they probably act as ongoing biomass carbon sinks on long-term timescales due to their low mortality rates and exceptionally long residence times that allow biomass to be accumulated for millennia.

## Introduction

Forest biomass is driven by the long-term balance between growth rate and mortality [1]. The conservation of large stocks of biomass in undisturbed forests avoids significant carbon emissions to the atmosphere, so biomass quantification in primary forests is crucial under current climate change policy debates [2]. Net primary productivity (NPP) corresponds to the total organic matter produced per unit time and is an important component of the global carbon cycle [3]. Changes in climate and atmospheric composition are likely to induce changes in NPP, therefore it is important to understand the magnitude, drivers and allocation of NPP in ecosystems [3].

NPP components include the production of leaves, stems, branches, coarse and fine roots, volatile organic compounds, root exudates, among others; however only the biomass components of this total production are usually quantified. A number of studies have assessed forest NPP, or more frequently aboveground NPP (NPP_AG_)_,_ especially in temperate and boreal ecosystems from the Northern Hemisphere (e.g. [4–6]) and in tropical systems (see [7] for a compilation). However, very few studies have examined productivity in southern hemisphere temperate forests, such as those in southern South America [8–10].

One of the most compelling and least well-understood tree species of the southern hemisphere is *Fitzroya cupressoides* (Molina) I.M. Johnst. (Cupressacea) or alerce, an evergreen conifer from southern South America that may reach 5 m in diameter and 50 m in height, and can live more than 3600 years [11,12]. This is the second longest-lived tree recorded in the world [12] and the longest-lived tree that forms dense, tall stands (the longest live tree known, the bristlecone pine (*Pinus longaeva* D.K. Bailey), occurs in very sparse densities in semi-arid regions). *Fitzroya* is a single species endemic to the temperate rainforests of Chile and Argentina and has a disjunct distribution between 39° 50' and 43° S along three distinctive areas: the Coastal Range of Chile (from ca. 550 to 1000 m a.s.l), the Andean Range of Chile and adjacent Argentina (mainly from ca. 500 to 1200 m a.s.l) and locally in the Chilean Central Depression at ~41° S (from 35 to 175 m a.s.l, [13,14]).

Due to the beauty and durability of its wood, *Fitzroya* has suffered a long history of exploitation since the European colonization in the 1500’s [11,15]. In 1976, this species was declared a Natural Monument and its exploitation was prohibited in Chile. However, the cutting and use of “dead *Fitzroya*” is still permitted if the trees were killed before 1976, a condition that has stimulated illegal cutting and intentional burning to obtain “dead wood” [16]. This species is currently listed as endangered in the IUCN Red list of threatened species [17]. *Fitzroya* covers an area of 258,371 ha, of which 18% are protected within National Protected Areas [18].

Research on the biomass and productivity of *Fitzroya* has previously been conducted only in the southern portion of the Coastal Range at Chiloé Island [9,10]. Reported values of aboveground NPP there varied between 4.6 and 6.9 Mg dry biomass ha^-1^ year^-1^, with large differences in terms of wood productivity; between 2.7 and 5.1 Mg ha^-1^ year^-1^ in the studies of [10] and [9], respectively. No studies have examined the carbon budget of the much older and higher biomass *Fitzroya* forests of the Andes, where year-long research is especially challenging because of difficult accessibility, adverse weather conditions and the high costs of sampling remote forests.

Because of the slow growth rates but long lifetime of *Fitzroya*, and its endemic and endangered status, it is interesting and important to assess the current ecological condition of these forests, focusing on their primary production. These forests are unique in terms of their longevity, so studies in these ancient temperate forests can potentially provide more general insights into the functioning and carbon cycle of old growth temperate ecosystems. Moreover, studies on these forests can contribute to the quantification of regional and more accurate global carbon budgets, improving the representation of southern hemisphere ecosystems and particularly of southern South America forests in global carbon studies [6].

The aim of this research is to primarily describe the biomass carbon dynamics of contrasting *Fitzroya* forests of medium and very old age growing in the northern portion of the Coastal Range and the Andean Cordillera of southern Chile, respectively. These two ranges contain *Fitzroya’s* main populations and forests that are highly contrasting in terms of disturbance regime, development stage and prevailing environmental conditions. The studied stands are representative of the mean adult (mature) forest structure in each of these ranges. Specifically, we examine and compare (i) aboveground biomass; (ii) aboveground biomass productivity NPP_AG_ and its allocation between wood and canopy tissue; (iii) total biomass NPP and its above- and belowground allocation and iv) woody biomass residence time and its variation between species. In order to provide general information about the studied forests in each area, we also characterize the forest structure and species composition in each site.

Questions we specifically address include:

1. How do stand structure and aboveground biomass vary between the two sites?
2. How do patterns of NPP and its allocation vary between the two sites?
3. How do the productivity and carbon dynamics of these forests compare to other temperate forest ecosystems worldwide?

## Methods

### Study sites

**Fig 1:**
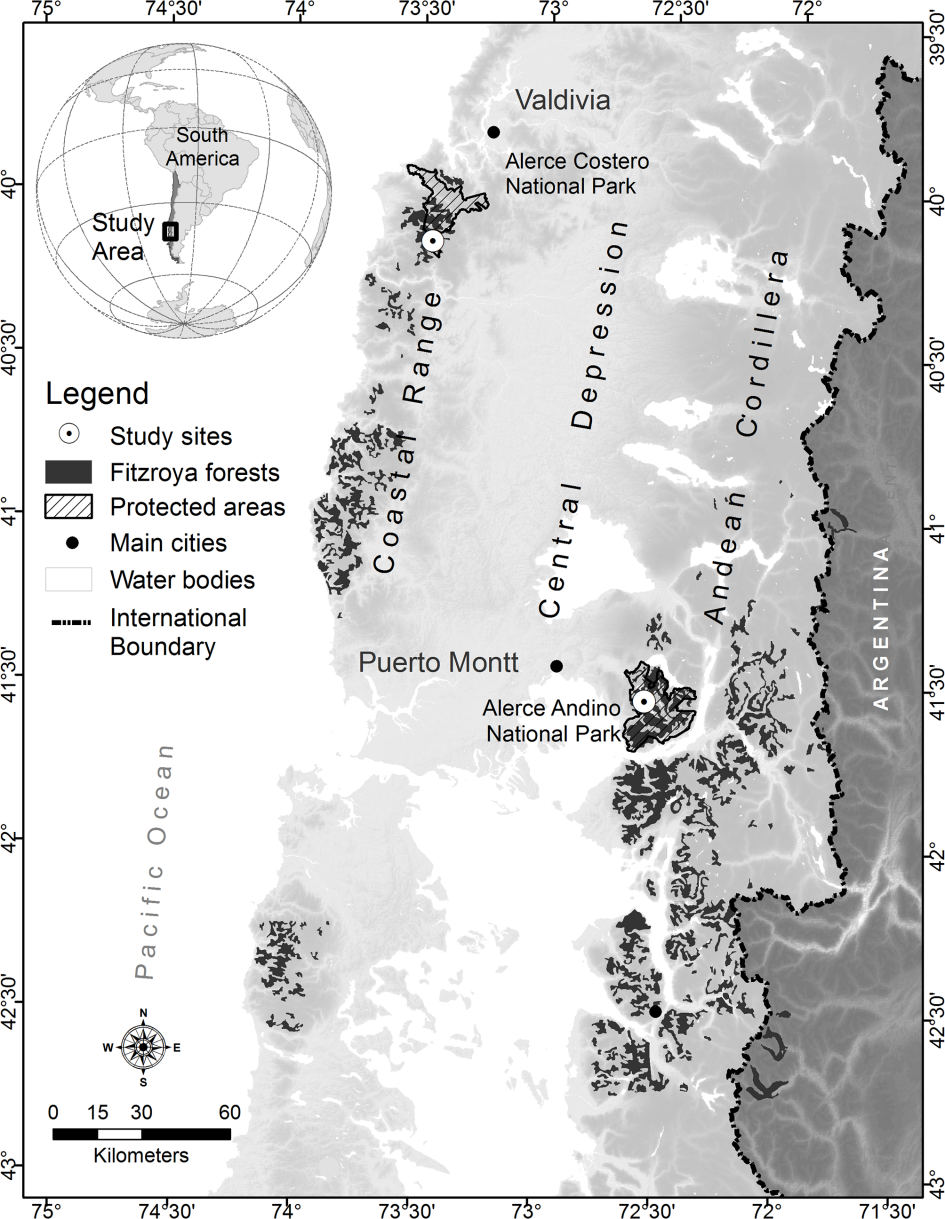
Location map of the study sites. Location map of the study sites in southern Chile, indicating the distribution of *Fitzroya* forests in Chile and Argentina. The map does not show stands located in the Central Depression near Puerto Montt due to their small area. Darker shaded areas correspond to higher altitudes in the Coastal Range and Andean Cordillera.

We worked with an endangered tree species within National Parks, so the Chilean National Forest Service "Corporación Nacional Forestal, CONAF" granted the permission to work and develop the following reported activities in *Fitzroya cupressoides* forests within the two involved National Parks. Two 0.6 ha plots (AC1 and AC2) were installed on very gentle slopes in the Alerce Costero National Park (AC) at 40° 10’ S- 73° 26’ W in July-August 2011 (Fig 1 and Figure A in S2 File.). This Park is located on the Coastal Range and the mean altitude of both plots is 850 m.a.s.l. Two similar 0.6 ha plots (AA1 and AA2) were installed on a southwest-facing slope (ca. 20%) in the Alerce Andino National Park (AA) at 41° 32’ S- 72° 35’ W (~ 760 m a.s.l) in August-October 2011 (Fig 1 and Figure A in S2 File.). Data analysed correspond to one year from November 2011 to October 2012.

*Fitzroya* forests in the Coastal Range have developed following frequent low to mid-intensity fires and the landscape is commonly characterized by stands formed by living trees mixed with snags from older cohorts [19,20]. Most of fires in recent centuries have been caused by humans, although lightning may also be an influence [20]. Forests in the Coastal Range have also been affected by historical harvesting and by recent illegal cuttings since the end of the 1970s. In the Andes, *Fitzroya* forests are normally characterized by old growth even-aged stands with large trees, and have developed following large-scale disturbances such as volcanic ash deposition, lava flows and landslides [21,22].

The Coastal Range was not affected by Pleistocene glaciations and the soils originate from Pre-Cambrian to Paleozoic metamorphic rocks. Soils in this area have a low pH, are generally thin, poor in nutrients and severely podzolized [23]. Climate is characterized by high annual precipitation and a mild temperature range, and according to a non-automated rain gauge in the park, mean annual precipitation was 4180 mm during 1999–2010 [24]. The region has a Mediterranean climate influence, with approximately 47% of the annual precipitation occurring in winter (June to August) and ca. 9% during summer (December to February, [24,25]). Fires have likely affected the study site during the last century [20,26].

The Andes at the study site latitude were extensively glaciated during the Pleistocene and most of the surfaces that were covered by ice at that time have been covered by recent andesitic volcanic deposits [11]. Soils where *Fitzroya* develops in the Andes are derived from volcanic parental materials, contain high organic matter, have high C/N ratios and low pH [27,28]. Climate conditions have not previously been described for the study area, but a nearby station at 240 m a.s.l (Lago Chapo ~18 km northeast of the study site) recorded a mean annual temperature of 10.3°C and 4140 mm of annual precipitation [12].

### Meteorological data

Automatic weather stations (Skye instruments, Powys, UK) were installed in clearings less than 1 km from the study plots in both areas. These stations recorded precipitation, relative humidity, temperature and total solar radiation every 30 minutes. Soil temperature was recorded with a Decagon sensor EC-T (Pullman, WA, USA) installed at a single point at 10 cm depth within the plots AC1 and AA1.

### Soil characterization

After testing soil conditions using a steel soil sampler, two and three soil pits were excavated in AC1 and AC2 plots, respectively for physical and chemical characterization. In the Andes two soil pits were excavated in each plot. Chemical analyses were done separately for each surface horizon, where most of the roots develop. Analyses included the main macro- and micronutrients and were performed at the Laboratorio de Suelos Forestales at Universidad Austral de Chile.

### Biomass and NPP_AG_ measurements

The main measurements in each 0.6 ha (60x100 m) plot were taken following and adapting the RAINFOR-GEM network protocol ([29], www.gem.tropicalforest.ox.ac.uk), where plots were subdivided in 15 subplots of 20x20 m.

Every tree ≥ 10 cm diameter at breast height (DBH, 1.3 m) was censused and tagged and its DBH measured. Bark thickness was measured in three points around the trunk of every *Fitzroya* tree with a handmade tool especially designed for this purpose. This is mainly because *Fitzroya*, especially when it is older, develops a deep and spongy bark.

There is only one biomass allometric equation available for *Fitzroya*, developed using a non-destructive method in Chiloé Island, and that uses only DBH as the independent variable [30]. Therefore, in order to have specific biomass estimates for each study site, volume equations were developed for each area. Volume was calculated with the Smalian’s formula for different trunk sections using diameter measured at different heights with a Spiegel Relaskop (Relaskop-Technik, Austria) and lengths of stems [31]. 35 and 40 trees were measured for this purpose in AC and AA, respectively. Different models relating volume with DBH and height were fitted [31] and the best equation was chosen according to goodness of fit and residual diagnosis. Two different volume equations, using diameter with and without bark were developed for each site and used for each tree.

An estimate of a biomass expansion factor (BEF) for *Fitzroya* was obtained using the volume of branches calculated with the diameter and length of all the branches up to 1 cm diameter in five trees fell in an illegal cutting. The affected stand was located in the Coastal Range further south from AC at 40° 38’ S, 73° 36’ W. Total volume (stem plus branches) was divided by stem volume in order to get the BEF. The estimated value for this factor was 1.066 (±0.01) and is the only existent approximation of this factor for the species. This value may not be that accurate for the Andean trees, since larger/older *Fitzroya* trees likely have larger and less branches, as well as a less symmetrical branch pattern than smaller trees [32]. Wood density was determined as the mean of three basic density measurements obtained through the water displacement method using three available cross sections from sites close to both study areas. This procedure was carried out at the Laboratorio de Maderas (Wood Laboratory) at Universidad Austral de Chile. Values for AC and AA were 0.411 (±0.008) and 0.444 (±0.015) g/cm^3^, respectively.

Volume without bark, multiplied by the BEF and wood density provided an estimate of biomass without bark per each tree. The difference between volumes with and without bark obtained for each tree and an available estimate of bark density from the Andean site (0.203 ±0.019 g/cm^3^), were used to calculate bark biomass. Total woody biomass for *Fitzroya* trees, was obtained through the sum of biomass without bark and the biomass of the bark.

For the other tree species, biomass equations already available for Chile were used [33–35]. When a biomass equation did not exist for certain species, another one from a species of the same family or with similar structural characteristics was used.

In order to have an estimate of woody biomass for trees < 10 cm DBH, two 10x10 m plots were established within each plot and small trees (2–10 cm DBH) were censused using callipers. The same equations mentioned above were used to estimate biomass.

Aboveground coarse wood productivity (NPP_*ACW*_) was assessed by determining the growth rate of existing surviving trees considering trees ≥ 10 cm DBH, as well as the smallest tree component (trees < 10 cm DBH) in the small plots scaled up to the hectare. A key difference from the normal protocol was that the slow growth rates and large tree diameters in *Fitzroya* made it difficult to measure annual growth by conventional forest inventories or even manual dendrometers. Instead we analysed tree rings to determine annual growth.

The estimate of the growth rate (mean growth for the last five years) was done by collecting two tree-ring cores from 15 *Fitzroya* trees per plot (across diameter classes) and from three trees per diameter class of the other species per plot. This sampling was restricted mainly because the plots are within National Parks and the National Forest Service (CONAF) established a maximum number of samples to extract in the associated permit. The mean growth rate per species was extrapolated to the trees that were not sampled. Tree-radial growth was added to diameter measurements and the new woody biomass was estimated with the allometric equations mentioned above. The difference in biomass corresponds to the annual woody productivity. Biomass and woody productivity calculations were made at a tree level in each plot and aggregated to the hectare.

Branch turnover productivity (NPP_*branch turnover*_) includes all woody material ≥ 2 cm diameter and was assessed by conducting censuses of fallen branches from live trees along two parallel 1x100 m transects in each plot, every three months.

Canopy productivity (NPP_*litterfall*_) was estimated collecting litterfall at a monthly basis in fifteen 0.25 m^2^ (50 cm x 50 cm) litterfall traps located approximately at the center of every 20x20 m subplot and placed at 1 m height above the ground. Litterfall from the understory was also collected in each site.

Further details on this methodology are provided in Methods in S1 File.

### Total biomass and productivity

Total aboveground productivity was calculated for the period November 2011-October 2012, by summing the above-mentioned components:

$$NPP_{AG}=NPP_{ACW}+NPP_{branchturnover}+NPP_{litterfall}$$ (1)

In a parallel paper, [36] estimated fine root productivity (NPP_*fine root*_) from a mass conservation approach balancing soil heterotrophic respiration with organic matter inputs (carbon inputs to the soil were assumed to be equal to carbon outputs, including any change in carbon stocks, [37,38]). This was applied at whole site level rather than plot level using the following equation:

$$NPP_{fineroot}=R_{h}-Litterfall-(Mort_{AG}+NPP_{branchfall})*F_{CWD}-Mort_{BG}+F_{DOC}+\Delta C$$ (2)

Where:

*R*_*h*_: soil heterotrophic respiration for the period November 2011-October 2012.

*Litterfall*: mean annual amount of litterfall collected in both plots from each site.

*Mort*_*AG*_: mean aboveground mortality.

*NPP*_*branchfall*_: mean productivity associated to this component

*F*_*cwd*_: fraction of coarse woody debris (CWD) entering the soil (not lost through in situ respiration).

*Mort*_*BG*_: belowground mortality which totally enters the soil.

*F*_*DOC*_: carbon leakage.

*ΔC*: net change in carbon stocks.

*F*_*DOC*_ and *ΔC* were assumed to be zero or insignificant terms on an annual basis. Values for the other parameters can be found in Table A in S2 File.

In addition, we estimated coarse root NPP (NPP_*coarse root*_) as a fixed fraction of NPP_*ACW*_, using the coarse root/aboveground biomass ratio found in *Fitzroya* forests from Chiloé (6.9%, [39]). This is the only estimate of this ratio available for these forests and we applied a conservative uncertainty estimate of ±75% for this parameter. Total NPP was calculated as the sum of NPP_AG_ plus the belowground components.

All estimated productivity values are reported in Mg of carbon (C) ha^-1^ year^-1^ (biomass values are reported in Mg C ha^-1^). To convert dry biomass into carbon, the mean carbon content of aboveground biomass in Chilean temperate species (49.64%) was used [40]. All reported errors consider ±1 SE.

Error propagation was carried out using standard rules of quadrature, assuming that uncertainties are independent and normally distributed (e.g. [41]).

### Mean wood residence time

This variable, an indicator of the mean time woody carbon remains in a system, was calculated using the most common approach as the ratio of mean standing woody biomass and mean woody productivity obtained through allometric equations [42]. In order to include only the main tree component, woody biomass and productivity included trees ≥ 10 cm DBH. This estimate must be considered an approximation, since this approach is valid just for systems where standing biomass stocks are near equilibrium. For aggrading systems residence time tends to be underestimated by this method; hence the calculated value is a minimum estimate for residence time.

## Results

### Climate and soil characterization

Precipitation was high year-round and moderately seasonal; total annual precipitation was 4450 mm in AC and ~6300 mm in AA (November 2011-October 2012). Mean annual air temperature was similar between sites, although lower in the Andes (7.5°C and ~ 7.1°C in AC and AA, respectively, Figure B in S2 File.).

In both sites soils are acidic, depths are ≤ 1 m and there is a dense fine root layer in the upper 30 cm (Table B in S2 File). Soils are sandy loam and silty loam in AC and AA, respectively. Soils in AC are shallower and much poorer in nutrients (especially nitrogen and cations) than soils in AA (Table B in S2 File). Nutrient poor soils in the Coastal Range site are associated with the old parental material (metamorphic rocks), that has experienced a persistent process of nutrient lixiviation [23]. The C/N ratio is high in both areas, indicating a low decomposition rate leading to carbon accumulation, but especially high in the Andes, probably due to wetter conditions and lower soil temperatures (Table B in S2 File). Finally, soils in both sites have a high exchangeable aluminium content. This feature indicates high toxicity for the roots, especially in AC, where the high aluminium content makes the limited nutrients even less available for the roots [43].

More details on climate and soil characteristics are presented in Results in S1 File.

### Question 1. How do stand structure and aboveground biomass vary between the two sites?

#### Forest composition and structure

Tree density (trees ≥ 10 cm DBH) was 1415 and 1408 stems ha^-1^ and basal area was 89 and 87 m^2^ ha^-1^ in AC1 and AC2, respectively. *Fitzroya* constituted around 85% of the tree stems in each plot and 93% and 94% of the total basal area in AC1 (83 m^2^ ha^-1^) and AC2 (82 m^2^ ha^-1^), respectively. The biggest trees were *Fitzroya*, which reached up to 86 cm DBH. The mean height of the canopy *Fitzroya* trees (≥30 cm diameter) was 14.4 m (±1.14) in AC1 and 14.1 m (±1.86) in AC2, with the maximum recorded height being 17.6 m in AC2. Stem volume for this species was 446 and 443 m^3^ ha^-1^ in AC1 and AC2, respectively (see volume equations in Table C in S2 File). The largest proportion of basal area in both plots was present in the 30–40 cm diameter class (Figure C in S2 File.).

The second most abundant species in both AC plots was the evergreen *Drimys winteri* (Winteraceae). Two species from the *Nothofagus* genus (*Nothofagus nitida* and *Nothofagus betuloides*, Nothofagaceae) that frequently hybridize [44] were especially common in AC2. Other evergreen broadleaved tree species such as *Weinmannia trichosperma* (Cunoniaceae), *Embothrium coccineum* (Proteaceae) and *Tepualia stipularis* (Myrtaceae), accompanied *Fitzroya*. The understory was dominated by *Chusquea montana* (Poaceae), small trees of *Embothrium coccineum*, and by other evergreen species, such as *Gaultheria mucronata* (Ericaceae), *Gaultheria insana*, *Ugni candollei* (Myrtaceae), *Desfontainia fulgens* (Columelliaceae) and the fern, *Blechnum magellanicum* (Blechnaceae). *Philesia magellanica* (Philesiaceae) was a rather widespread epiphyte commonly growing on *Fitzroya* trunks.

At the Andean site, stem density (trees ≥ 10 cm DBH) was lower at 782 and 720 trees ha^-1^ in plots AA1 and AA2, respectively and *Fitzroya* accounted for just 12% and 13% of this total number. However, *Fitzroya* accounted for 79% and 81% of total basal area, which reached 171 and 193 m^2^ ha^-1^ in AA1 and AA2, respectively. The largest tree was 235 cm DBH in AA2. The mean heights of *Fitzroya* trees were 31.80 m (±4.31) and 30.85 m (±4.82) in AA1 and AA2, respectively, with the maximum height recorded being 45.7 m in AA1. Stem volume for this species reached 1474 and 1777 m^3^ ha^-1^ in AA1 and AA2, respectively. More than half of the trees were distributed in the smallest diameter class (10–20 cm DBH) and the contribution of each size class to basal area was more or less stable up to 70 cm and increased significantly from 100 cm DBH due to the presence of *Fitzroya* (Figure D in S2 File.). A notable feature was the almost complete absence of *Fitzroya* trees < 80 cm DBH, their domination of size categories > 100 cm, and the presence of trees with extremely large diameters. This condition, coupled with its slow growth rate, indicate that there has not been any significant recruitment of *Fitzroya* for several centuries.

*Fitzroya* was mostly accompanied by evergreen broadleaved species of which the most abundant were *Nothofagus nitida* (Nothofagaceae) and *Myrceugenia chrysocarpa* (Myrtaceae). Other species included *Laureliopsis philippiana* (Monimiaceae), *Amomyrtus luma* (Myrtaceae) and two evergreen conifers *Podocarpus nubigenus* (Podocarpaceae) and *Saxegothaea conspicua* (Podocarpaceae). *Saxegothaea* was especially abundant in both plots. The understory was dominated by *Chusquea montana*, *Desfontainia fulgens*, *Blechnum magellanicum* and *Philesia magellanica*.

The number of small trees (2–10 cm DBH) reached 1800 and 3050 trees ha^-1^ in AC1 and AC2, respectively, a high number already considering the density of trees ≥10 cm DBH. The main tree species represented in both plots was *Drimys winteri*.

The number of trees < 10 cm DBH was 950 and 1600 trees ha^-1^ in AA1 and AA2, respectively. The main tree species represented in both plots was *Saxegothaea conspicua*.

#### Aboveground biomass

**Fig 2:**
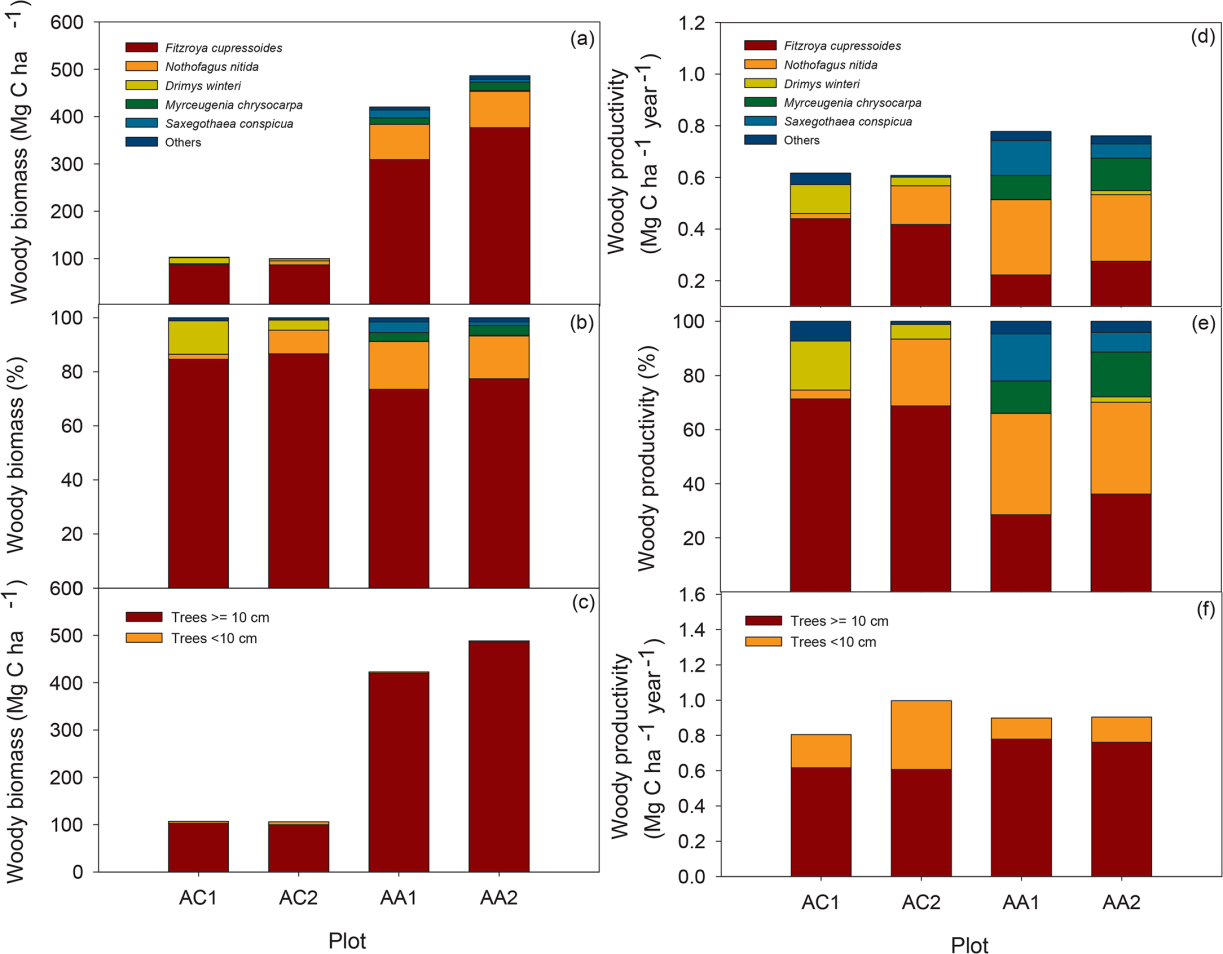
Aboveground woody biomass and productivity per species and plot. Left panel: a) Aboveground woody biomass per species in all trees ≥10 cm diameter, b) proportion of the woody biomass presented in a) contributed by the different species in percentage (%) and c) woody biomass contributed by the large (trees≥10 cm DBH) and small (trees < 10 cm DBH) tree components within each plot. Right panel: d), e), f) the same as a), b) and c), respectively, but for aboveground woody productivity.

The total aboveground woody biomass was 107.0 and 105.8 Mg C ha^-1^ in AC1 and AC2 respectively, with 3.77% and 5.49% of this in trees < 10 cm DBH. Total biomass was much greater in the Andes (423.1 and 488.5 Mg C ha^-1^ in AA1 and AA2 respectively), with trees < 10 cm DBH only accounting for 0.64 and 0.45% (Table 1, Fig 2). *Fitzroya* dominated biomass in both study sites with more than 70% of the total biomass. *D*.*winteri* and *Nothofagus spp*. were the second most important in biomass in AC1 and AC2, respectively. In the Andes, *N*. *nitida* was the second most important (Fig 2).

**Table 1:** Aboveground biomass and productivity per plot.

	Alerce Costero (AC)				Alerce Andino (AA)			
	AC1		AC2		AA1		AA2	
Component	Mean	SE	Mean	SE	Mean	SE	Mean	SE
Woody biomass (≥ 10 cm) **(**Mg C ha^-1^)	103.0	10.3	100.0	10.0	420.4	42.0	486.3	48.6
Woody biomass (< 10 cm) **(**Mg C ha^-1^)	4.0	0.4	5.8	0.6	2.7	0.3	2.2	0.2
Canopy biomass (≥ 10 cm) **(**Mg C ha^-1^)	6.2	0.6	6.0	0.6	23.9	2.4	28.2	2.8
Canopy biomass (< 10 cm) **(**Mg C ha^-1^)	0.18	0.02	0.25	0.03	0.13	0.01	0.09	0.01
Understory biomass **(**Mg C ha^-1^)	0.70	0.15	0.70	0.15	0.35	0.13	0.35	0.13
**Total biomass (**Mg C ha^-1^)	**114.1**	10.3	**112.8**	10.0	**447.5**	42.1	**517.1**	48.7
NPP_*ACW*_ (≥ 10 cm) (Mg C ha^-1^ year^-1^)	0.62	0.12	0.61	0.12	0.78	0.16	0.76	0.15
NPP_*ACW*_ (< 10 cm) (Mg C ha^-1^ year^-1^)	0.19	0.04	0.39	0.08	0.12	0.02	0.14	0.03
Total NPP_*ACW*_ (Mg C ha^-1^ year^-1^)	0.81	0.13	1.00	0.14	0.90	0.16	0.90	0.15
NPP_*branch turnover*_ (Mg C ha^-1^ year^-1^)	0.039	0.01	0.038	0.01	0.15	0.05	0.40	0.22
NPP_*litterfall*_ (Mg C ha^-1^ year^-1^)	2.27	0.33	2.16	0.31	1.09	0.06	1.16	0.09
NPP_*litterfall understory*_ (Mg C ha^-1^ year^-1^)	0.23	0.07	0.16	0.02	0.08	0.02	0.08	0.02
**Total NPP**_**AG**_ (Mg C ha^-1^ year^-1^)	**3.35**	0.36	**3.36**	0.34	**2.22**	0.18	**2.54**	0.28
Total NPP_fine root_ (Mg C ha^-1^ year^-1^)	0.81	0.60	0.81	0.60	1.50	0.42	1.50	0.42
Total NPP_coarse root_ **(**Mg C ha^-1^ year^-1^)	0.06	0.04	0.07	0.05	0.06	0.05	0.06	0.05
**Total NPP** (Mg C ha^-1^ year^-1^)	**4.21**	0.70	**4.24**	0.69	**3.78**	0.46	**4.10**	0.51

Total woody, canopy and understory biomass, yearly aboveground coarse wood productivity (NPP_*ACW*_), canopy productivity (NPP_*litterfall*_), branch turnover productivity (NPP_*branch turnover*_) and total aboveground productivity (NPP_AG_) for one year of data for Alerce Costero (AC1 and AC2) and Alerce Andino plots (AA1 and AA2). Estimates of fine root productivity (NPP_*fine root*_) from [36], coarse root productivity (NPP_*coarse root*_) and total NPP calculated in this study are also shown. SE is standard error of the mean

**Fig 3:**
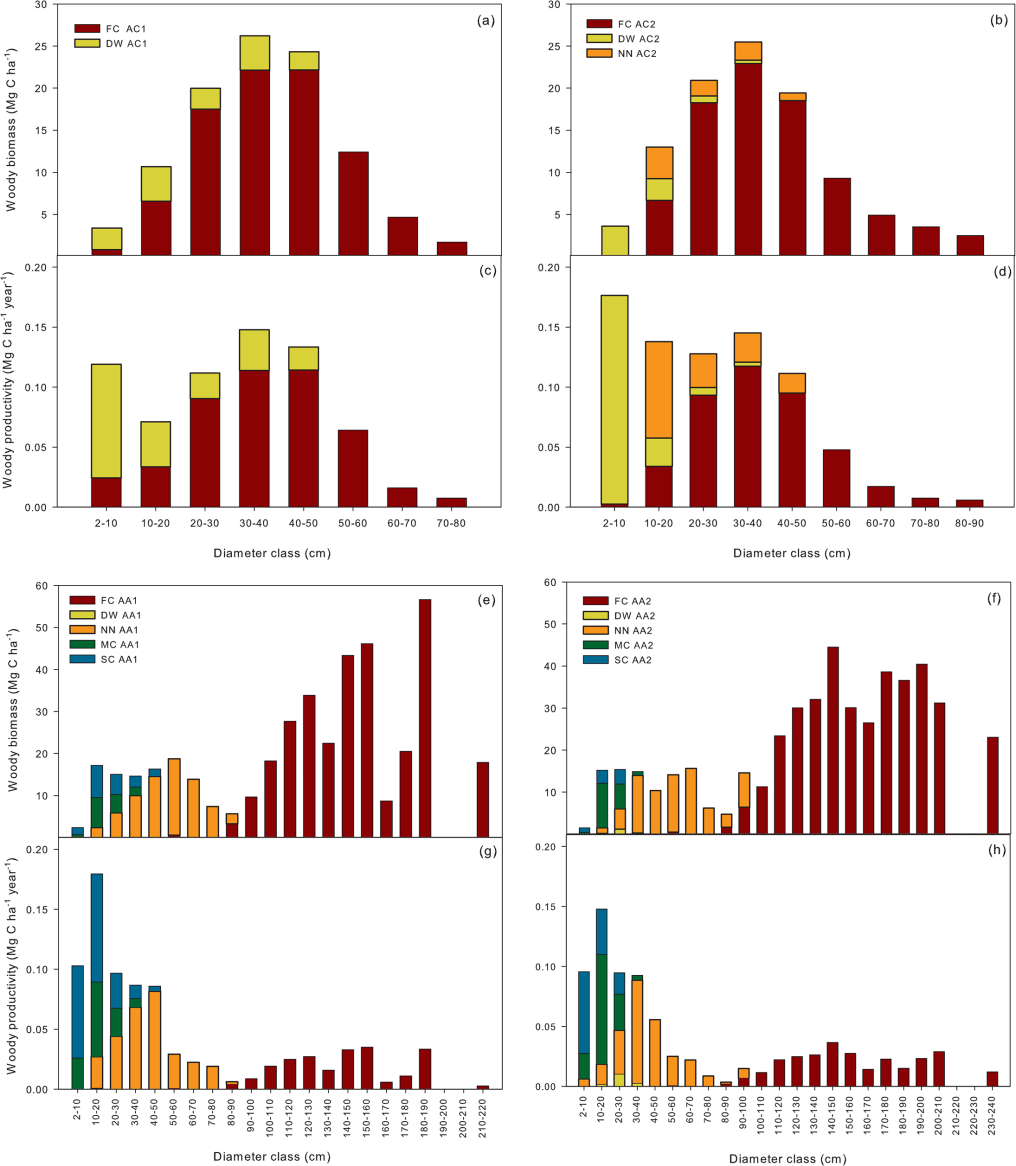
Aboveground woody biomass and productivity per species and diameter classes. a), b), e), f) aboveground woody biomass for each of the most important species along different diameter classes in Alerce Costero and Alerce Andino plots. c), d), g), h) the same as in a), b), e), f) respectively, but for aboveground woody productivity. FC: *Fitzroya cupressoides*, NN: *Nothofagus nitida*, DW: *Drimys winteri*, MC: *Myrceugenia chrysocarpa*, SC: *Saxegothaea conspicua*.

Most of the woody biomass in AC was mainly distributed between 30 and 50 cm DBH (Figure E in S2 File.). *Fitzroya* was dominant throughout the diameter distribution in AC, except below 10 cm DBH, where *D*.*winteri* dominated biomass. In AA, *Fitzroya* entirely accounted for the very large diameter classes, while *N*.*nitida* mostly contributed to biomass between 30 and 90 cm, and *S*.*conspicua* and *M*.*chrysocarpa* at lower diameter classes (Fig 3).

Total canopy biomass reached 6.4 and 6.3 Mg C ha^-1^ in AC1 and AC2, respectively. 2.81 and 4.01% of this amount corresponded to trees < 10 cm (Table 1). Estimated canopy biomass reached 24.0 and 28.3 Mg C ha^-1^ in AA1 and AA2, respectively. 0.54 and 0.33% of this value corresponded to trees < 10 cm (Table 1).

Total aboveground biomass was 114.1 and 112.8 Mg C ha^-1^ in AC1 and AC2, respectively, while total biomass in AA reached 447.5 and 517.1 Mg C ha ^-1^ (Table 1). Total biomass from the understory was 0.61–0.62% and 0.07–0.08% of these values in AC and AA, respectively.

### Question 2. How do patterns of NPP and its allocation vary between the two sites?

#### Aboveground productivity

Total NPP_*ACW*_ was 0.81 and 1.00 Mg C ha^-1^ year^-1^ in AC1 and AC2, respectively, with 23 and 39% accounted for by trees < 10 cm DBH. In the Andean site, total aboveground woody productivity was 0.90 Mg C ha^-1^ year^-1^ in both plots, with 13 and 16% accounted for by small trees < 10 cm DBH (Table 1, Fig 2). In AC there was no recorded mortality (trees ≥ 10 cm DBH) in the census interval and in AA1 and AA2 respectively, two and four non-*Fitzroya* trees ≥ 10 cm DBH died (0.20%/0.09% of woody biomass, Figure F in S2 File.). *Fitzroya* was the species that, among trees ≥10 cm DBH, mostly contributed to productivity in AC and was the second most important after *N*.*nitida* in AA1 (Fig 2).

An important proportion of total woody productivity appeared concentrated in the smallest tree component (< 10 cm DBH) especially in AC2 (Fig 3 and Figure E in S2 File.). In larger diameter classes, most of productivity concentrated in the 30–50 cm diameter class in AC and in the 10–20 cm diameter class in AA (Fig 3 and Figure E in S2 File.). In AC most of woody productivity in the smallest diameter classes (< 20 cm DBH) can be attributed to *D*.*winteri* and *N*.*nitida*. In AA most of productivity below 20 cm can be attributed to *S*. *conspicua* and *M*.*chrysocarpa* and to *N*.*nitida* between 30 and 90 cm. *Fitzroya* is the sole contributor above 100 cm DBH (Fig 3).

Total annual branchfall (NPP_*branch turnover*_) was almost the same in AC1 and AC2 and higher in AA, with more than double in AA2 than in AA1 (Table 1). Most of the branches, at least in the Andean site, fell during the winter period, probably because of windstorms or snow loads on branches (June-August; Figure G in S2 File.).

Mean annual fine litterfall (NPP_*litterfall*_ above 1 m) for the studied period was 2.27 ±0.33 and 2.16 ± 0.31 Mg C ha^-1^ year^-1^ in AC1 and AC2, respectively (Table 1). Litterfall reached 1.09 ± 0.06 and 1.16 ± 0.09 Mg C ha^-1^ year^-1^ in AA1 and AA2, respectively; values significantly lower than the AC site (Table 1).

Litterfall from the understory (below 1.0 m) added an additional 10.1% and 7.4% to annual litterfall from vegetation above 1 m in AC1 and AC2, respectively. These values were 7.3% and 6.9% in AA1 and AA2, respectively (Table 1). For more details about litterfall seasonality and production see Results in S1 File and Figures H and I in S2 File.

### Total NPP

NPP_AG_ was 3.35 ± 0.36 and 3.36± 0.34 Mg C ha^-1^ year^-1^ in AC1 and AC2 and 2.22 ± 0.18 and 2.54 ± 0.28 in AA1 and AA2, respectively (Table 1). Total NPP was 4.21±0.70 and 4.24±0.69 Mg C ha^-1^ year^-1^ in AC1 and AC2 and 3.78±0.46 and 4.10±0.51 Mg C ha^-1^ year^-1^ in AA1 and AA2, respectively (Table 1).

**Fig 4:**
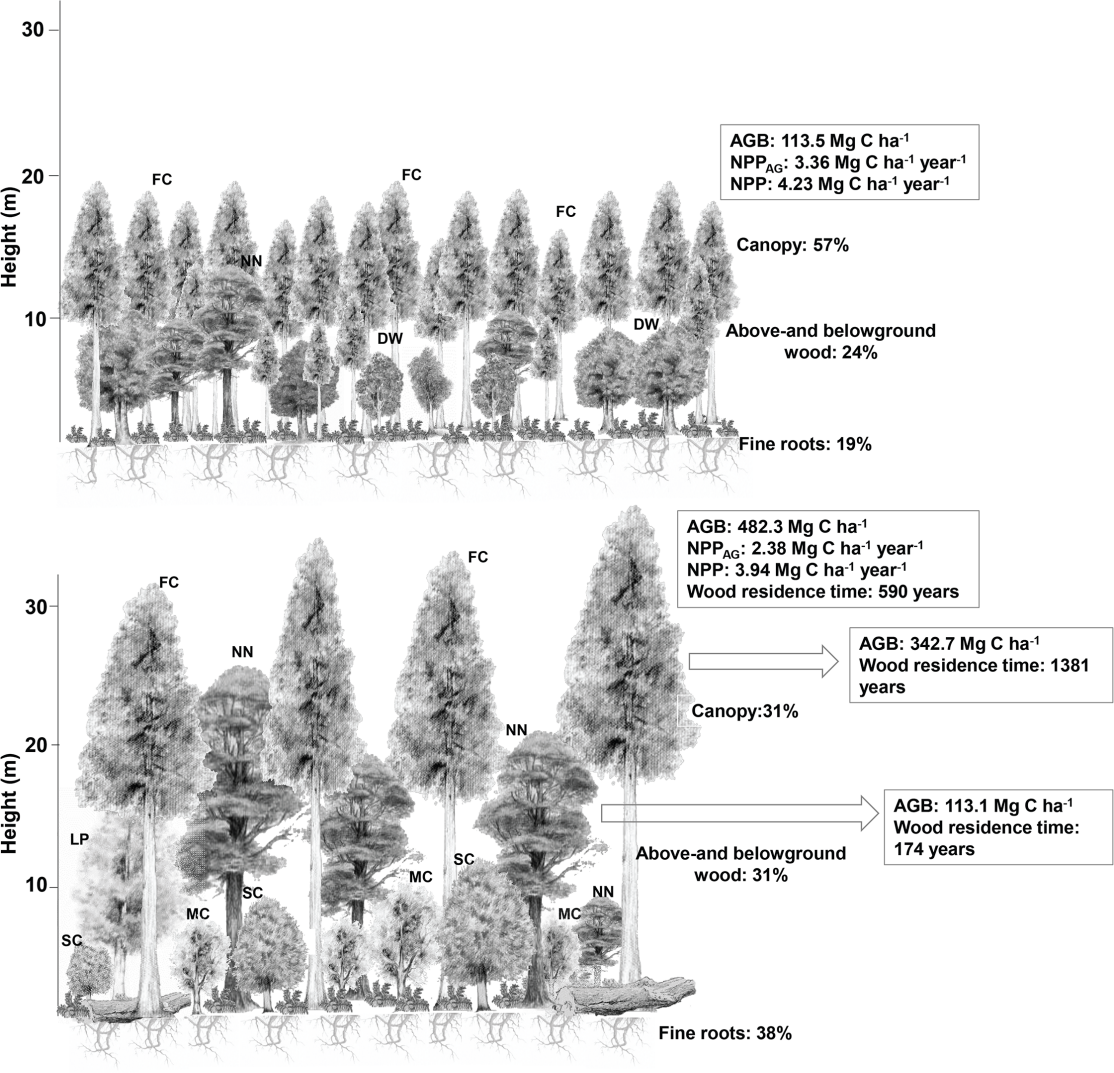
Forest structure and carbon dynamics in Alerce Costero and Andino. Diagram exemplifying the structure of the forest in the coastal (upper panel) and the Andean site (lower panel). The main species in each forest are identified, the mean values for each carbon cycle component from both plots and the productivity allocated to canopy, wood and fine roots (in %) are shown. Arrows indicate separated values for the *Fitzroya* stand only and the *Nothofagus* dominated subcanopy forests in AA. FC: *Fitzroya cupressoides*, NN: *Nothofagus nitida*, DW: *Drimys winteri*, LP: *Laureliopsis philippiana*, MC: *Myrceugenia chrysocarpa*, SC: *Saxegothaea conspicua*. AGB: aboveground biomass, NPP_AG_: aboveground productivity, NPP: total productivity.

**Fig 5:**
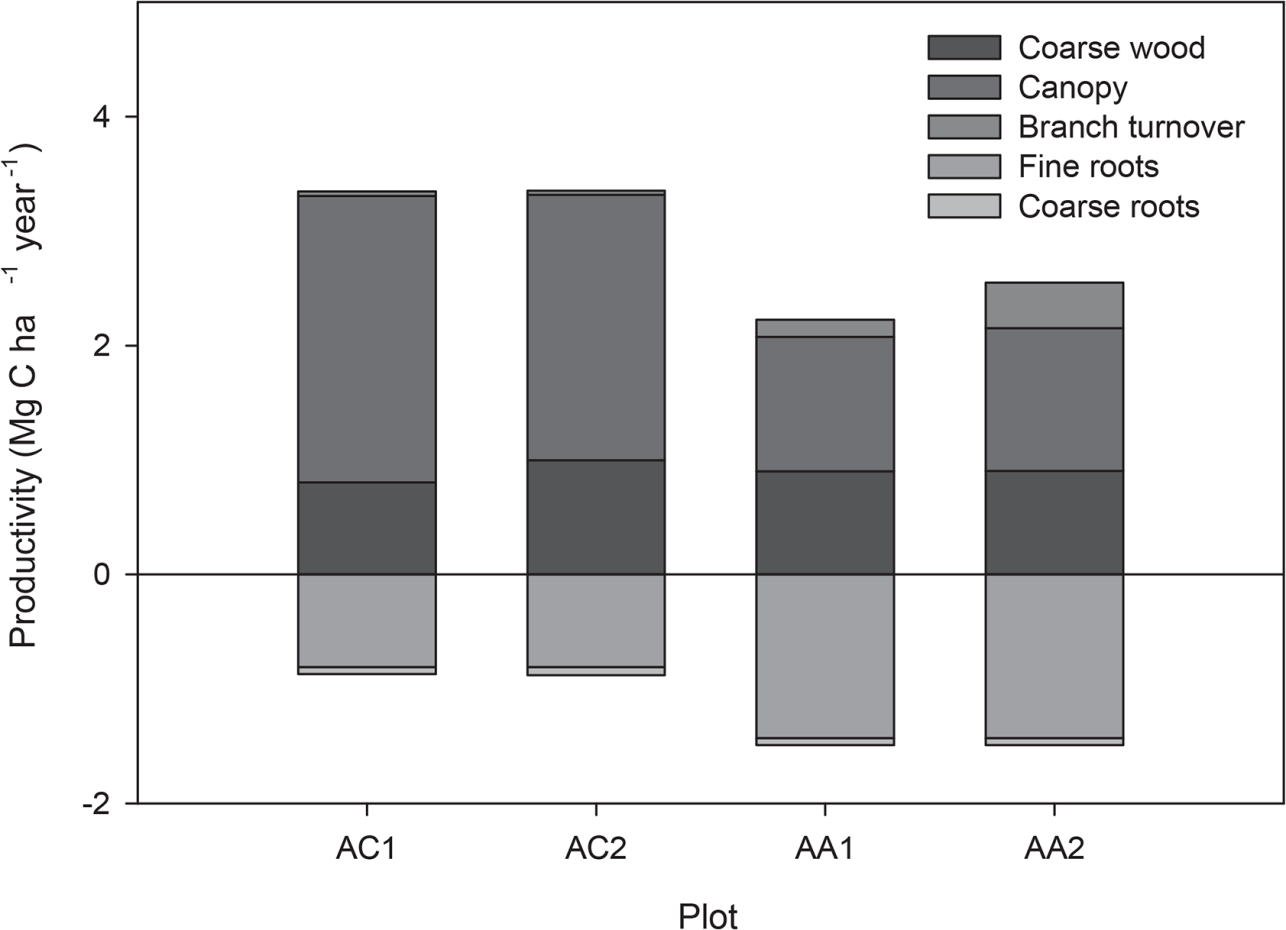
Total productivity per plot. Total productivity in Mg C ha^-1^ year^-1^ for the one-year period November 2011-October 2012 in the four studied plots and its allocation to the different components. Below-ground NPP is indicated as negative values.

In the AC sites, on average 24% of NPP was allocated to woody production (including coarse roots), 19% to fine roots, and 57% to canopy. In the AA sites, 31% of NPP was allocated to woody production (including coarse roots), 38% to fine roots, and 31% to canopy (Fig 4). Hence, total NPP was somewhat similar in both sites, but in the younger site, more was allocated to canopy production and less to fine roots, with woody allocation being closer in both sites (Fig 5).

Canopy productivity was higher than total aboveground wood productivity (*NPP*_*ACW*_ + *NPP*_*branchturnover*_) in all sites, except AA2 (Table 1). However, branchfall is dominated by episodic events, and it seems likely that high branchfall in AA in one year is not representative of longer-term branch turnover in the old-growth forest.

### Mean wood residence time

**Fig 6:**
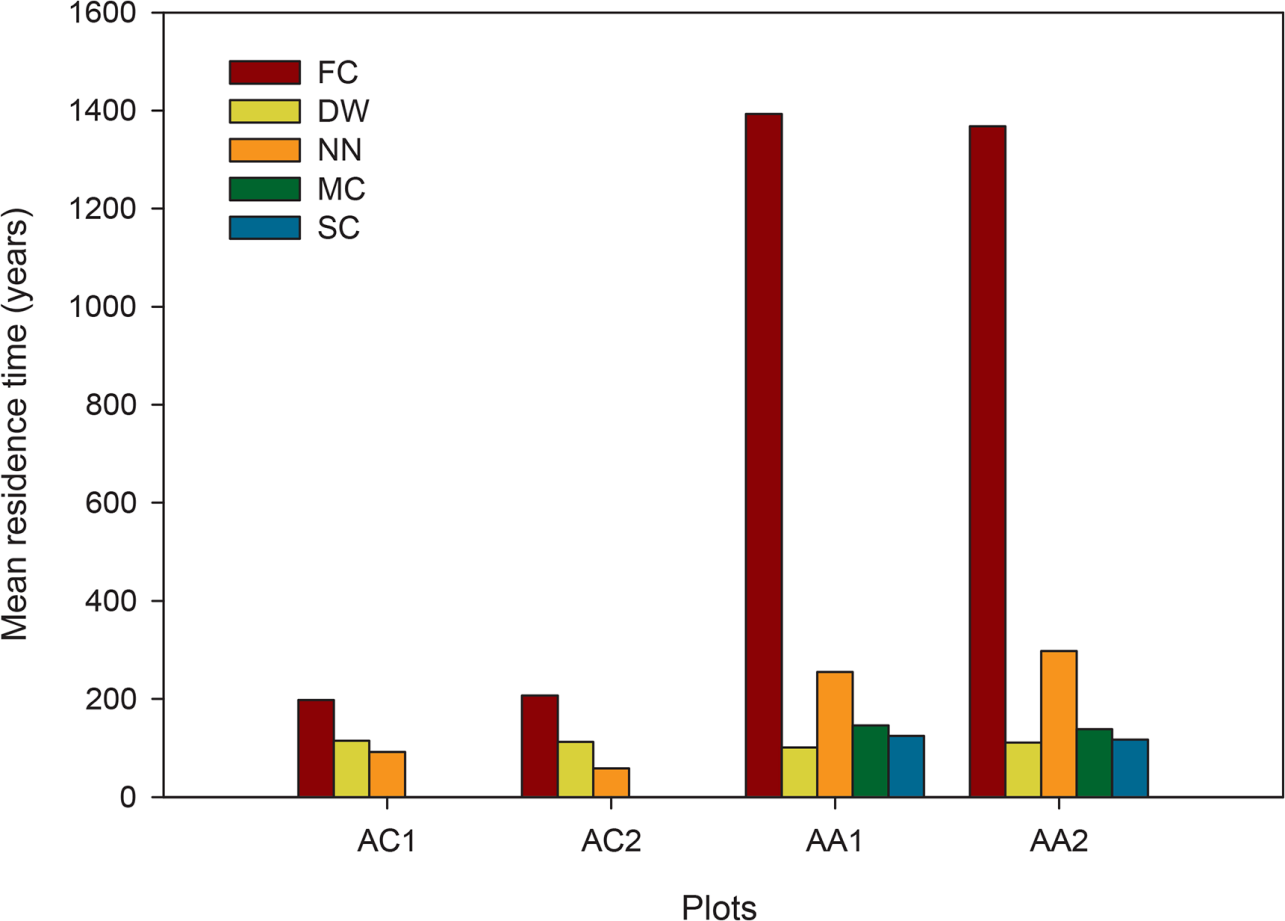
Mean wood residence time per species and plot. Mean wood residence time for the main species in each plot. FC: *Fitzroya cupressoides*, NN: *Nothofagus nitida*, DW: *Drimys winteri*, MC: *Myrceugenia chrysocarpa*, SC: *Saxegothaea conspicua*.

The mean wood residence time (for trees ≥ 10 cm DBH), calculated by dividing aboveground wood biomass by coarse wood productivity, was 166 ± 36 and 164 ± 36 years in AC1 and AC2, respectively. Estimates were 539 ± 123 and 640 ± 142 years in AA1 and AA2, respectively. When we considered the *Fitzroya* trees alone, they had mean residence time values of 198 ± 44 and 207 ± 46 years in AC1 and AC2 and of 1393 ± 311 and 1368 ± 306 years in AA1 and AA2, respectively. *Nothofagus nitida* stood out in the Andes as the species with the second longest residence time (average value of 277 ± 62 years, Fig 6). The long residence times estimated for *Fitzroya* in AA are consistent with the minimum age of some trees in the stand (1200–1470 years old) derived from the tree ring data collected in the area.

Estimates of residence time are underestimates in the case of AC, as no mortality was recorded during the studied period, indicating that the forest stands have not reached quasi-equilibrium where mortality biomass loss equals woody biomass production. In AA there is not much difference between NPP_ACW_ and mortality, because of the large *Nothofagus* trees (20–64 cm DBH) that died during the one-year interval (Figure F in S2 File.). However, we recorded no mortality in the dominant *Fitzroya* in AA and hence this species, like the forest in AC, may still be experiencing long-term biomass accumulation. The Andean site appears characterised by a very old *Fitzroya* canopy that is still accumulating biomass, and a *Nothofagus*-dominated “sub-canopy” that is closer to dynamic equilibrium, with mortality balancing growth. In addition, trunks and snags of *Fitzroya* take many decades or even centuries to decompose. We did not observe any recently dead *Fitzroya* trunks at either AA plot, implying that there has been no mortality for probably several decades or centuries.

## Discussion

*Fitzroya* forests in the Andes and in their northern distribution in the Coastal Range appear to be very different in terms of structure and species composition, but there has not been information until now on how different these forests can be in terms of biomass and productivity. This study provides for the first time combined estimates of aboveground biomass, NPP and carbon allocation in stands growing in these two areas.

### Forest structure, biomass and productivity of *Fitzroya* forests in the study sites

Differences in forest structure between plots from the same site were slight, indicating the homogeneity of the studied forest sites. The difference in forest structure between AC and AA is mainly due to different disturbance regimes in both areas. Due to the approximate age of the oldest trees in AC (~ 300 years old) and the presence of some large diameter snags, it is plausible to assume that the studied forest was established after a fire occurred in 1681 [20,26]. There are almost no particularly large trees and old-growth forests in the Coastal Range, due to fires and forest cuts since the 1500s [23]. In the Andes plots, by contrast, *Fitzroya* is mostly present in large diameter classes and there is little regeneration as it is a relatively shade intolerant species [15]. In such a forest, the lack of small and medium *Fitzroya* trees indicates that most regeneration took place after a major disturbance (probably a landslide) over a thousand years ago. Similar distributions, characterized by an over-representation of large size-classes and restricted regeneration, have been reported for other shade-intolerant species mostly dependent on large-scale disturbance for recruitment [45]. Moreover, the high basal area of *Fitzroya*, its long-lived character and its pioneer and emergent status, suggests that these forests would be an example of the “additive basal area” phenomenon [45]. With their large diameters but small crowns, they would exert only limited shade on the remainder of the forest below, acting essentially as giant slow-growing “poles” contributing large amounts of biomass, but having only moderate influence on light competition and ensuing effects on forest dynamics.

In AA, the *Nothofagus*-dominated forest below the *Fitzroya* canopy has a biomass of 112–114 Mg C ha^-1^, woody productivity of 0.63–0.68 Mg C ha^-1^ year^-1^ and woody biomass residence times of 169 ± 38–178 ± 40 years. These values compare very well with biomass, productivity and residence times values found in evergreen *Nothofagus* forests in New Zealand (Table 2, 210 years of residence time, [46]) and with biomass values in montane mixed-broadleaf evergreen forests in Chiloé Island [10] and evergreen *Nothofagus* forests in Tierra del Fuego, Argentina ([47], Table 2). This would be consistent with the suggestion that the *Fitzroya* stand does not have a major influence in perturbing the productivity and dynamics of the *Nothofagus* forest that sits below. However, further studies on *Nothofagus* forests growing under similar conditions would be needed to draw firmer conclusions.

**Table 2:** Aboveground biomass and productivity in temperate forests worldwide.

Forest^a^	Site	AGB	NPP_*ACW*_	NPP_*litterfal*_	NPP_AG_	Reference
		(Mg C ha^-1^)	(Mg C ha^-1^ year^-1^)	(Mg C ha^-1^ year^-1^)	(Mg C ha^-1^ year^-1^)	
*Fc*	Coastal Range, Chile (AC)	112.8–114.1	0.81–1	2.32–2.5	3.35–3.36	This study
*Fc*	Andean Cordillera, Chile (AA)	447.5–517.1	0.9	1.17–1.24	2.22–2.54	This study
*Fc*	Chiloé Island, Chile	285.9	2.53	0.89	3.42	[9,39]
*Fc*	Chiloé Island, Chile	268.4	1.34	0.94	2.28	[10]
*Fc*	Coastal Range, southern Chile	NA	NA	1.62	NA	[48]
*Fc*	Chiloé Island, Chile	NA	NA	1.01	NA	[49]
*Mb*	Chiloé Island, Chile	116.4	2.28–2.78	1.64–3.77	4.42–6.06	[9,10]
*Ac*	El Bolson, Argentina	78.4–99.9	1.05–1.4	1.7–2.3	2.95–3.6	[8]
*Nb*	Tierra del Fuego, Argentina	105–156	2.05–3.36	NA	NA	[47]
*Aa*	Trounson Reserve, N. Auckland	729	1.20	2.05	3.25	[50]
*Ns*	Craigieburn Range, N. Zealand	122.6	0.59	2.03	2.62	[46]
*Ps-Th*	Western Coast Range, Oregon	355.4	4.15	1.1	5.25	[51]
*Pm-Th*	Oregon Cascades	781	NA	NA	NA	[52]
*Ap-Pm*	Oregon Cascades	440	4.9	1.6	6.5	[52]
*Tm-Al-Pe*	High Cascades summit, Oregon	NA	NA	NA	2.55	[53]
*Pe-Al*	Rocky Mountain N.P. Colorado	126.5	0.97	0.89	1.86	[54]
*Ss*	Humboldt Redwoods State Park	366–1650	NA	NA	2.65–9.40	[55]
*Ss*	Humboldt Redwoods State Park	1928–2320	2.5–3.5	0.5–2.5	3.5–5	[56]
*EN*	Pacific Northwest	228.4	NA	NA	7.35	[57]
*MOT*	Worldwide	5.45–1650	0.58–9.4	0.3–3.45	1.67–11.34	[58]
*OT*	US Pacific Northwest	464.7	NA	NA	NA	[59]
*MOCTM*	Worldwide	377	NA	NA	NA	[2]

^a^: Fc: Fitzroya cupressoides, Mb: Mixed-broadleaf evergreen forests, Ac: Austrocedrus chilensis, Nb: Nothofagus betuloides, Aa: Agathis australis (values reported are just for this species and not for the accompanying trees), Ns: Nothofagus solandri, Ps-Th: Picea sitchensis-Tsuga heterophylla, Pm-Th: Pseudotsuga menziesii-Tsuga heterophylla, Ap-Pm: Abies procera-Pseudotsuga menziesii, Tm-Al-Pe: Tsuga mertensiana-Abies lasiocarpa-Picea engelmanii, Pe-Al: Picea engelmanii-Abies lasiocarpa, Ss: Sequoia sempervirens, EN: Evergreen needleleaf, MOT: Mature-old-growth temperate forests, OT: Old-growth temperate forests, MOCTM: Mature-old-growth cool temperate moist forests

Estimates of aboveground biomass (AGB), aboveground coarse wood productivity (NPP_*ACW*_), canopy productivity (NPP_*litterfall*_) and total aboveground productivity (NPP_AG_) for different temperate forests worldwide. Values reported in Mg of dry biomass/productivity in the original studies were converted to Mg of C using the coefficient 0.5 in the case of studies in other countries and the same coefficient as in this study in the case of forests in Chile

Our calculation of around four times more aboveground biomass in AA than AC constitutes a first estimate of the difference between forests growing in these two Ranges. This demonstrates the carbon storage capacity of *Fitzroya* forests under undisturbed conditions and in areas affected by recurrent fires. Biomass calculations may be underestimates, however, due to the low BEF estimated for *Fitzroya* from field measurements (1.066). This factor was low compared with mean values reported for other conifers (e.g. 1.18–1.21, [60]), but it was the only approximation available for the species. On the other hand, the underestimate might be somewhat counteracted by the fact we do not consider losses by heart rot due to lack of information.

The low woody productivity values reported in this study are indicated by the low radial growth rates found for all the species and especially *Fitzroya*. Mean annual rates for *Fitzroya* were 0.22 and 0.31 mm year^-1^ in AA and AC, respectively and for most of the other species, this rate never surpassed 1 mm year^-1^. Reported mean growth rates for *Fitzroya* range from 0.28 to 2.99 mm year^-1^ [13], so values in this study are at the lower end of these ranges. Moreover, woody productivity was an important component in small diameter classes (DBH < 10 cm) especially in AC2, mainly due to the likely overestimation of woody biomass caused by using allometric equations just with DBH as the independent variable and the high density of these small trees.

The slow growth rate observed in the diverse species growing in these sites may be mainly explained by the low nutrient conditions of the soils. Traits related to nutrient retention, as opposed to traits related to growth, appear to be more relevant for the dominance of certain tree species in low fertility sites in the Coastal Range of southern Chile [61]. In the case of the Andean site, low radiation associated to the very high precipitation that falls in this area cannot be discarded as an additional driver of the slow growth rate of tree species.

When considering aboveground components, overall NPP_AG_, and particularly canopy productivity, was lower in AA than AC, probably due to the different development stage of these forests. Older forests have been generally reported to be less productive than younger forests [62–64]. However, when belowground components were also included, we found little difference in NPP between the sites, suggesting that the observed differences in NPP_AG_ may be more driven by differences in allocation than in productivity. It is interesting to note that allocation to fine roots was not high in the Coastal Range site, where very poor soil conditions are prevalent. This may be due to the high exchangeable aluminium content that prevents cell division and root elongation [65], suggesting that root allocation is not in proportion to nutrient limitation. Fine root biomass in this site is around twice that in the Andes [36], implying that fine root residence time is longer in this area.

It has also been reported that foliage production decreases with age in conifer forests, which is consistent with our observations in the year reported here [66,67]. However, a subsequent year of litterfall measurements (data available online), provided almost the same amount in the Andes, but half the amount in the Coastal Range. Both sites experienced lower rainfall and warmer temperatures during the second summer (41% and 31% reduction in rainfall during January and February in AC and AA, respectively), but only in AC this did result in lower soil moisture status, suggesting that interannual variation in productivity is much higher in the younger site.

### Question 3. How do the productivity and carbon dynamics of these forests compare to other temperate forest ecosystems worldwide?

#### Comparison with other forests in Chile and worldwide. Forest structure and biomass

The mean parameters found in AC (3215 and 4458 stems ha^-1^ and 92.5 and 91.4 m^2^ ha^-1^ considering trees ≥ 2 cm DBH in AC1 and AC2, respectively) are different from the ones reported for *Fitzroya* forests in Chiloé. The density of the Chiloé forests was 9680 trees ha^-1^ (trees ≥ 2 cm) and the basal area was 138.2 m^2^ ha^-1^, a very high value considering that *Fitzroya* trees are somewhat similar in size to the ones in AC [39].

Aboveground biomass values in AC (112.8–114.1 Mg C ha^-1^) are smaller than the value reported for old-growth evergreen forests close to Valdivia (140 Mg C ha^-1^, [68]) and lower than the ones reported for *Fitzroya* forests in Chiloé ([39], Table 2). In these Chiloé forests, more than the presence of large trees, tree density determines the high biomass reported.

Looking beyond Chile, the biomass values in AC are smaller than the mean reported for evergreen needleleaf forests from the US Pacific North West ([57], Table 2) and higher than the mean reported for secondary *Austrocedrus chilensis* forests in Argentine Patagonia (67.1 Mg C ha^-1^ and 156.8–199.8 Mg dry mass ha^-1^, [8,69], Table 2). Biomass values in AC are also higher than the value reported by the IPCC for Temperate Oceanic forests of South America (180 Mg dry mass ha^-1^, [70]).

On the other hand, the biomass values in AA (448–517 Mg C ha^-1^) are higher than all the mentioned values above and higher than the one reported for an old-growth *Nothofagus* dominated forest in the Andes further north (435 Mg C ha^-1^, [71]). Looking at other high biomass temperate forests, these biomass values are also higher than the mean compiled for mature and old growth cool temperate moist forests in a worldwide database ([2], Table 2) and the value in AA2 higher than the highest mean value reported for old-growth forests in the US Pacific northwest ([59], Table 2). Cool temperate moist forests as a biome; hold the highest mean value of aboveground biomass per unit area worldwide [2], locating *Fitzroya* forests from the Andes as one of the most massive forests in the world. The biomass values reported here for AA are amongst the highest reported for coniferous species in the world. The other forests with high biomass values are *Tsuga heterophylla*, *Pseudotsuga menziessi*, *Pseudotsuga-Tsuga* (all Pacific Northwest), *Sequoia sempervirens* (California) and *Agathis australis* (New Zealand), although this last estimate was based on limited sampling ([2,50,52,55,56,72], Table 2). The only forest stands with substantially higher biomass are *Sequoia* in California. However, *Fitzroya* is unique amongst the large conifers in combining such massive biomass with the lowest radial growth rate and productivity, implying that its exceptional longevity has a particularly important role in determining the high biomass of these stands. The longevity and slow growth rate of *Fitzroya* compared with other long-lived tree species growing in much drier sites was recognized by [73] in his work “Longevity under adversity in conifers”. The adversity in the sites where *Fitzroya* lives may be mainly given by the poor soil nutrient conditions and by low radiation which may limit photosynthesis in these very rainy areas.

#### Aboveground productivity per component

Woody productivity values in both sites are lower than the values reported for *Fitzroya* and mixed-broadleaf evergreen forests in Chiloé [9,10], and are also at the lower end of values reported for mature temperate forests in a global database ([58], Table 2).

Litterfall values in AC are a little higher than the mean reported for a *Fitzroya* site close to the study area [48] and much higher than the values reported for *Fitzroya* forests in Chiloé [9,10,49] (Table 2). Values are however in the range reported for mixed-broadleaf evergreen forests in this island (Table 2).

Litterfall values in AC are within the range of values reported for warm temperate needleleaf evergreen forests (~3.3 to 6.9 Mg dry biomass ha^-1^ year^-1^, [74]), but values in AA, fall below this range. Values in both sites are, on the other hand, within the range of measured values for mature temperate forests ([58], Table 2).

#### Total productivity, carbon allocation and mean wood residence time

Total NPP_AG_ values in AC (3.35–3.36 Mg C ha^-1^ year^-1^) are in the range reported for *Fitzroya* forests in Chiloé by [9] and [10] (Table 2). The values in AA (2.22–2.54 Mg C ha^-1^ year^-1^) approximate the one reported by [10], where lower litterfall production is compensated by higher woody productivity. However, studies in Chiloé do not include branchfall. Finally, values in both study sites are lower than total NPP_AG_ reported for mixed-broadleaf evergreen forests in Chiloé (Table 2).

Our NPP_AG_ estimates suggest that *Fitzroya* forests, especially in the Andes, have low productivity compared with other temperate wet forests. NPP_AG_ in the Andes for example is lower than the value reported for stands of another long-lived species, *Agathis australis* (Table 2). Total NPP_AG_ in both sites are within the range, but at the lower end of values reported for mature temperate forests worldwide [58] and they are lower than values reported for conifer forests in the US Pacific North West ([51,52,57], Table 2). NPP_AG_ in both sites are on the range or higher though, than values reported for some high elevation forests in that country ([53,54], Table 2). Furthermore, the values in AA are lower than the lower bound NPP_AG_ estimate for old-growth *Sequoia* forests (Table 2).

Most of NPP_AG_ was allocated to the canopy, especially in AC. In contrast, most other temperate forests appear to allocate more to stem rather than foliage productivity [58,75]. However, other mature southern hemisphere forests (*Nothofagus* and *Agathis* forests from New Zealand and *Austrocedrus* forests from Argentine Patagonia), also presented lower stem compared with foliage productivity ([8,46,50], Table 2). When allocation between aboveground components was separated by species (something not possible for below-ground allocation), most of carbon in *Fitzroya* was allocated to the canopy (81% in AC and 68% in AA) rather than wood (19% in AC and 32% in AA). In the case of other species, most of carbon was allocated to wood (69–72% in AC and 58–59% in AA) rather than the canopy (28–31% in AC and 41–42% in AA). Patterns are more extreme in the Coastal Range site and overall results demonstrate that the slow-growing *Fitzroya* has a low priority for allocation to wood. A recently reported positive relationship between woody productivity and allocation to stem growth, albeit for tropical forests, might help explain this lower woody allocation in *Fitzroya*. Increased allocation to stem growth, might be a way for forests that are more productive to obtain faster growth rates [42]. Trees in the sub-canopy, in particular those with broad crowns such as *Nothofagus*, would experience more intense competition for light, and hence may be expected to prioritise woody growth to overtop competitors, over leaf canopy growth.

The overall low woody production in these sites compared with others may be explained by the low nutrient availability that characterize them, since wood production per unit photosynthesis seems to be lower at sites with these characteristics [76]. A lower biomass production per unit photosynthesis in nutrient poor soils may be associated with higher photosynthate investment in often non-accounted root symbionts [76]; vesicular-arbuscular mycorrhizae have been found for *Fitzroya* and other associated tree species in the Coastal Range [77,78].

Few temperate sites report total NPP. Our estimates of total NPP are lower than other reported for temperate forests in the Pacific Northwest: 6.8 Mg C ha^-1^ year^-1^ for an old-growth *Picea-Tsuga* forest and 7.9 Mg C ha^-1^ year^-1^ for evergreen needleleaf forests ([51,57], respectively). The Coastal Range forest site allocated a higher proportion of productivity aboveground (79%) compared with the Andean forest (59–62%). This finding is supported by the general notion that younger, aggrading forests allocate more carbon aboveground because of intense competition for available light, but does indicate that the poor soil conditions in AC do not increase biomass production to roots.

Finally, total mean wood residence time in AA was considerably higher than the values estimated for other old-growth and mature temperate forests in the US Pacific Northwest and New Zealand (85 to 292 years, [46,51,62,72]). Among the reviewed studies, the only ecosystem that slightly surpassed the Andean stand was the forest investigated by [56], ca. 717 years), which was dominated by *Sequoia* (> 80% of the number of trees). However, when we compare the *Fitzroya* trees separately, their residence times in AA greatly exceed those reported for other large forest stands.

Fig 4 depicts an example of the forest structure in each site summarizing the main carbon cycling estimates for these forests.

## Conclusions

We have described and contrasted the carbon stocks, productivity and residence times of two *Fitzroya* sites. The results show that these slow-growing forests show a non-equilibrium structure and dynamics hundreds of years after a disturbance, and *Fitzroya* may even continue to show a non-equilibrium structure and accumulate biomass more than a thousand years due to the lack of any observed mortality. The Andean *Fitzroya* forests are amongst the highest biomass forest sites in the world and *Fitzroya* has perhaps the longest residence time ever observed in the world. Forests in the Coastal Range do not reach such high biomass values, mainly due to fires in the past, but probably have the potential to do so over centuries if appropriate conservation is given to the species in this area. Although *Fitzroya* forests only represent ca. 2% of the total native forest cover in Chile and a negligible portion of the temperate forests in the southern hemisphere, these ecosystems are unique in the combination of the amount of biomass carbon and the length of time it can be stored due to their longevity and protected status. This fact gives support for their consideration in national and international carbon mitigation initiatives, as well as for their adequate protection against fires and illegal cuttings. It is important to increase the enforcement of the law that declared *Fitzroya* a National Monument in 1976, as well as to set long term restoration programs to recover *Fitzroya* in the Coastal Range and Central Depression where extensive areas have been burned in the last centuries.

*Fitzroya* stands are likely to be ongoing biomass carbon sinks at centuries to millennia timescales, due to their low mortality rates and particularly long wood residence times. We encourage future research to further understand the ecosystem ecology and dynamics of these unique and ancient forest ecosystems.

## Supporting Information

S1 File Supplementary methods and results. (DOC)

S2 File Supplementary tables and figures. Parameters to estimate fine root productivity **(Table A)**. Soil characteristics in both study sites **(Table B)**. Volume equations for *Fitzroya cupressoides* in each site **(Table C)**. Pictures of the studied forests **(Figure A).** Climate conditions in the study sites **(Figure B).** Diameter distribution and basal area in Alerce Costero **(Figure C).** Diameter distribution and basal area in Alerce Andino **(Figure D).** Total woody biomass and productivity per diameter classes and plot **(Figure E).** Aboveground coarse woody productivity and mortality per species and plot **(Figure F).** Seasonal cycle of branchfall per plot **(Figure G).** Seasonal cycle of litterfall and its components per plot **(Figure H).** Litterfall per component per plot **(Figure I).**
(DOCX)
