# Supplementary material for: The Oldest, Slowest Rainforests in the World? Massive Biomass and Slow Carbon Dynamics of Fitzroya cupressoides Temperate Forests in Southern Chile
Source: PLoS One. 2015 Sep 9;10(9):e0137569. doi: 10.1371/journal.pone.0137569 (PMC4564186; doi:10.1371/journal.pone.0137569)
Supplement: S1 File — (DOC) [file pone.0137569.s001.doc]

**Supplementary methods and results**

**Methods**

**Biomass stocks**

According to the RAINFOR-GEM network protocol [1] a one-ha plot is commonly used to assess biomass and productivity. However, it was not possible to fit such a plot in the AC site, since there were no accessible forest patches of this size in the Park, and 0.6 ha plots were used instead. Similar 0.6 ha paired plots were used in AA, to facilitate comparison and consistency between the two sites.

Heights were measured in at least 10% of the number of individuals per diameter class (10 cm classes) per species using a vertex IV (Haglof, Sweden). Heights for the rest of the trees were estimated when possible using a function that best fitted the data for each species and provided the expected normality and heterocedasticity in the residuals.

For non-*Fitzroya* tree species, when heights were effectively estimated through a robust height-DBH function (true in most cases), biomass equations that included height were used. In the few cases where the height-DBH relationship was not strong (e.g. *Tepualia stipularis, Myrceugenia spp., Maitenus magellanica, desfontainia fulgens, Amomyrtus luma*) and in the case of trees < 10 cm DBH, biomass equations using only DBH were used. Since the biomass equations considered all the aboveground components, published values for the mean proportion of the tree occupied by bole and branches (all components except leaves) were used to the get woody biomass for each species [2]. Total woody biomass of the plot was calculated by summing the biomass per hectare of all trees 2-10 cm DBH and ≥ 10 cm DBH.

Canopy biomass in each plot was estimated using the mean proportion of biomass present in this component published for different species [2]. In the case of *Fitzroya*, the mean proportion found between foliage biomass and biomass in stem and branches using the equations developed by [3] was used in this study.

Finally, a limited assessment of biomass present in the understory was undertaken through sampling of the four corners of a random 20x20 m subplot from one plot in AC and one in AA. 1x1 m quadrats were established and all vegetation below one meter height was cut and brought to the laboratory to determine dry weight. Since this component was sampled in one plot per site, the same mean value was assumed for both plots.

**Aboveground coarse wood productivity (NPP*_ACW_*)**

A small correction in productivity estimates was required for trees ≥ 10 cm that died within a year. It was assumed that trees that died did so on average half way through the census interval and then half of their growth was still considered as a contribution to productivity.

**Branch turnover productivity (NPP*_branch turnover_*)**

NPP*_branch turnover_* represents a branch turnover term not usually captured in NPP measurements [4], though it may have some bias if old trees are senescing and shedding branches.

Each branch turnover productivity transect was subdivided in five 20 m sub transects, corresponding to the sides of each 20x20 m subplot. Particular attention was paid to account only for branches coming from live trees. When a new branch was found within a sub transect, it was lifted and cut when needed, to only include the portion within the transect. Branches collected in each sub transect were classified in decomposition categories and weighed in the field. A representative sample (e.g. at least half the number of branches or even all branches) per decomposition category and sub transect was weighed wet and brought immediately to the lab to determine its dry weight and the corresponding water content. This water content was later used to determine the dry weight of all the samples collected in the field. Branchfall from transects was extrapolated to one hectare.

**Canopy productivity (NPP*_litterfall_*)**

Litterfall collection started in August and September 2011 in AC and in November 2011 in both Andean plots. Once collected, litterfall was immediately brought to the laboratory, separated into different components (leaves, needles, reproductive material, twigs and others), dried at 60 °C for 48 hours and weighed after removal from the oven. In order to assess litterfall from the understory vegetation, five litterfall traps per plot were installed just above the ground surface, in the four corner’s subplots and the center of each plot in AC. As the Andean site understory vegetation was less abundant, these traps were installed only in AA1. A second fine 1x1 m mesh was located at 1 m above the ground litter trap to exclude litterfall falling directly from above. These traps were installed in April 2012 and the first collection was made in May 2012. Therefore, monthly values from November 2012 to April 2013 had to be considered to obtain an annual estimate for this component. Since only one plot was sampled in AA, the mean value obtained for AA1 was extrapolated to AA2.

Total NPP*_litterfall_* was calculated adding litterfall collected from trees and from the understory.

**Fine root productivity (NPP*_fine root_*)**

Table A in S2 File shows the main parameters used to estimate fine root productivity according to the mass balance approach presented in the main manuscript.

**Results**

**Climate characterization**

Due to technical problems, meteorological data in the Andean site for May and most of June 2012 could not be recovered, so in order to have annual estimates gap filling was performed. Gaps were filled using linear regressions between monthly data at the study site and Puerto Montt (41° 25’ S, 73° 05’ W at 85 m a.s.l). This was done for precipitation, temperature and relative humidity using the period November 2011-October 2012.

There is a strong seasonality in solar radiation and temperature, with highest values observed during spring-summer (November to February) and the lowest values in fall-winter (May to August, Figure B in S2 File). Solar radiation was significantly lower at the Andean site (on average 22% lower) and soil temperature showed similar variation as air temperature, being consistently lower in the Andes (Figure B in S2 File). Highest precipitation values were recorded during fall-winter, but there was also a secondary peak in February 2012 (summer).

During winter months there was snow precipitation in both sites, particularly in the Andes where snow accumulates and lasts for many days. Relative humidity showed less marked seasonality in both sites with lower values in spring and summer.

**NPP*_litterfall_***

Total litterfall and needle production in AC and AA resembled each other in terms of seasonality (Figure H in S2 File). For other litterfall components, seasonality was somewhat different in both sites due to a more diverse species composition in the Andes. In both sites, peaks in litterfall were in late summer (February) and late autumn (May, Figure H in S2 File).

Litter fractions consisted of 90% and 83% *Fitzroya* needles, 6% and 12 % broadleaves, 1% reproductive material and 3% and 4% twigs in AC1 and AC2, respectively. Litter fractions for AA1 and AA2 corresponded to 43% and 50% *Fitzroya* needles, 44% and 38% broadleaves, 4 and 5% reproductive material and 9 and 7% twigs (Figure I in S2 File).

**References**

1. Marthews TR, Metcalfe D, Malhi Y, Phillips O, Huaraca Huasco W, et al. Measuring tropical forest carbon allocation and cycling: A RAINFOR-GEM field manual for intensive census plots (v2.2). 2012. Available: [www.gem.tropicalforest.ox.ac.uk](http://www.gem.tropicalforest.ox.ac.uk/). Accessed 25 April 2013.

2. Gayoso J, Guerra J, Alarcón D. Contenido de carbono y funciones de biomasa en especies nativas y exóticas. Report FONDEF Project N. D98I1076. Universidad Austral de Chile, Valdivia, Chile; 2002.

3. Vann DR, Palmiotto PA, Richard Strimbeck G. Allometric equations for two South American conifers: Test of a non-destructive method. For Ecol Manage. 1998; 106: 55-71.

4. Malhi Y, Aragão LEOC, Metcalfe DB, Paiva R, Quesada CA, et al. Comprehensive assessment of carbon productivity, allocation and storage in three Amazonian forests. Global Change Biol. 2009; 15: 1255-1274.
