# Supplementary material for: The Oldest, Slowest Rainforests in the World? Massive Biomass and Slow Carbon Dynamics of Fitzroya cupressoides Temperate Forests in Southern Chile
Source: PLoS One. 2015 Sep 9;10(9):e0137569. doi: 10.1371/journal.pone.0137569 (PMC4564186; doi:10.1371/journal.pone.0137569)
Supplement: S2 File — Parameters to estimate fine root productivity (Table A). Soil characteristics in both study sites (Table B). Volume equations for Fitzroya cupressoides in each site (Table C). Pictures of the studied forests (Figure A). Climate conditions in the study sites (Figure B). Diameter distribution and basal area in Alerce Costero (Figure C). Diameter distribution and basal area in Alerce Andino (Figure D). Total woody biomass and productivity per diameter classes and plot (Figure E). Aboveground coarse woody productivity and mortality per species and plot (Figure F). Seasonal cycle of branchfall per plot (Figure G). Seasonal cycle of litterfall and its components per plot (Figure H). Litterfall per component per plot (Figure I). (DOCX) [file pone.0137569.s002.docx]

**Supplementary tables and figures**

**Table A.** Parameters used to estimate NPP*_fineroot_* through the mass balance approach presented in the manuscript. Mean values for both plots in each site plus/minus standard errors are presented.

| Site | Rh  (Mg C ha^-1^ yr^-1^) | Litterfall  ( Mg C ha^-1^ yr^-1^) | Mort_AG_  ( Mg C ha^-1^ yr^-1^) | NPP_branchfa_ll  (Mg C ha^-1^ yr^-1^) | F_cwd_ | Mort_BG_  ( Mg C ha^-1^ yr^-1^) |
| --- | --- | --- | --- | --- | --- | --- |
| AC | 3.23±0.56 | 2.41±0.220 | 0 | 0.038±0.008 | 0.25±0.25 | 0 |
|  |  |  |  |  |  |  |
| AA | 2.99±0.33 | 1.21±0.064 | 0.66±0.33 | 0.270±0.11 | 0.25±0.25 | 0.045±0.041 |

**Table B.** Soil characteristics and nutrient composition in the upper horizons at plots AC1 and AC2 at the Alerce Costero site and plots AA1 and AA2 at the Alerce Andino site. Values shown are the averages of the soil profiles obtained in each plot

| Plot | Effective Soil depth^a^  (cm) | Surface horizon depth  (cm) | Bulk density  (g/cm^3^) | pH | SOM^b^  % | C/N | N  (%) | P (Olsen)  (ppm) | K  (ppm) | Ca  (ppm) | Mg  (ppm) | Na  (ppm) | Al^c^  (ppm) |
| --- | --- | --- | --- | --- | --- | --- | --- | --- | --- | --- | --- | --- | --- |
| AC1 | 63 | 23 | 0.75 | 4.09 | 9.57 | 34.5 | 0.17 | 3.1 | 72 | 100 | 71 | 28 | 715 |
| AC2 | 43 | 16 | 0.70 | 4.11 | 10.06 | 32.7 | 0.18 | 3.1 | 94.3 | 114.3 | 63.3 | 25.3 | 893 |
| AA1 | 72 | 18 | 0.16 | 4.23 | 78.70 | 113.2 | 0.40 | 5.2 | 470 | 510 | 204 | 94 | 410 |
| AA2 | 94 | 20 | 0.22 | 4.32 | 59.99 | 83 | 0.43 | 7.2 | 553 | 700 | 239 | 97 | 303 |

a: Soil depth where roots can potentially develop and extract water and nutrients without any apparent physical or chemical restriction.

b: Soil organic matter

c: Exchangeable Al

**Table C.** Volume equations used for *Fitzroya* trees in Alerce Costero (AC) and Alerce Andino (AA). Statistics associated to the equations are also presented

| Site | V_(withoutbark)_^a^ | R^2b^ | RSE‡ | DW¶ | V_(withbark)_^a^ | R^2b^ | RSE^c^ | DW^d^ |
| --- | --- | --- | --- | --- | --- | --- | --- | --- |
| AC | 0.00023*DBH^2.17614^ | 0.96 | 0.1910 | 1.46 | 0.00022*DBH^2.18308^ | 0.96 | 0.1936 | 1.41 |
| AA | 0.00008001*DBH^2.4823^ | 0.99 | 0.2131 | 1.59 | 0.00006877*DBH^2.5071^ | 0.99 | 0.2102 | 1.58 |

a: V_(withoutbark)_ : volume without bark in m^3^, V_(withbark)_: volume with bark in m^3^. DBH is in cm.

b: Adjusted R^2^

c: Residual standard error

d: Durbin Watson statistic. Residuals were not significantly autocorrelated according to this statistic.

| 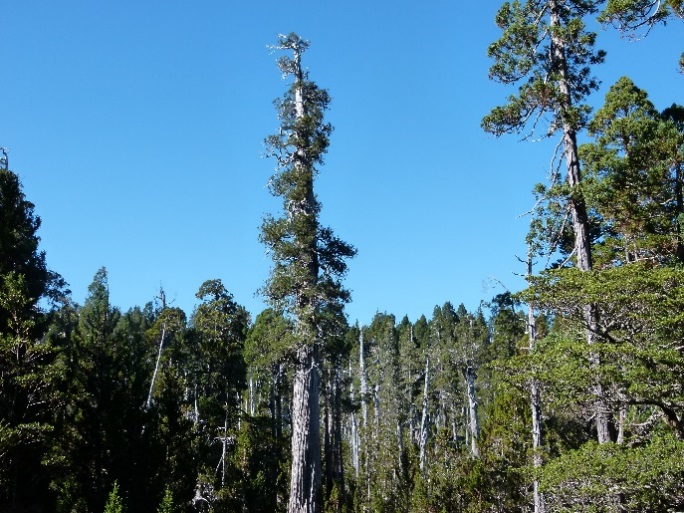 | 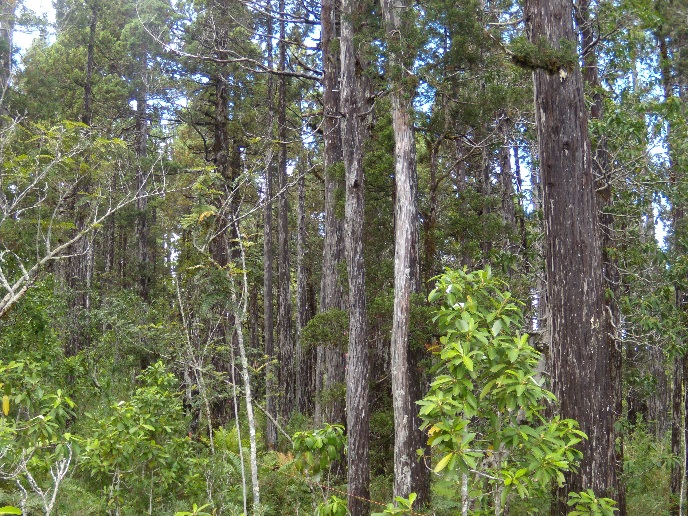 |
| --- | --- |
| 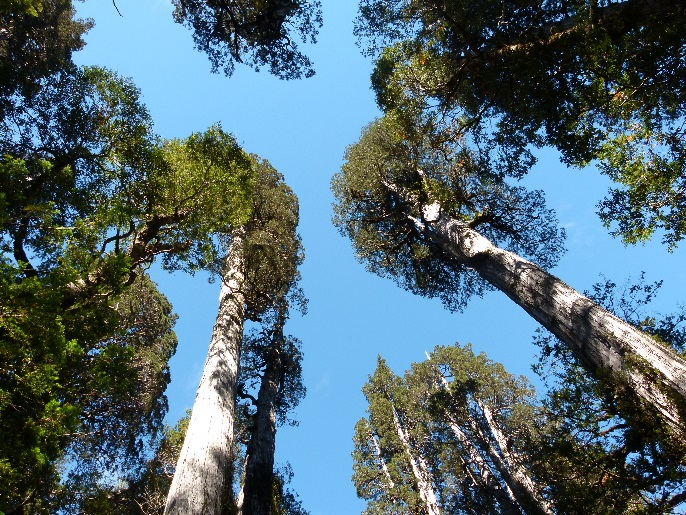 | 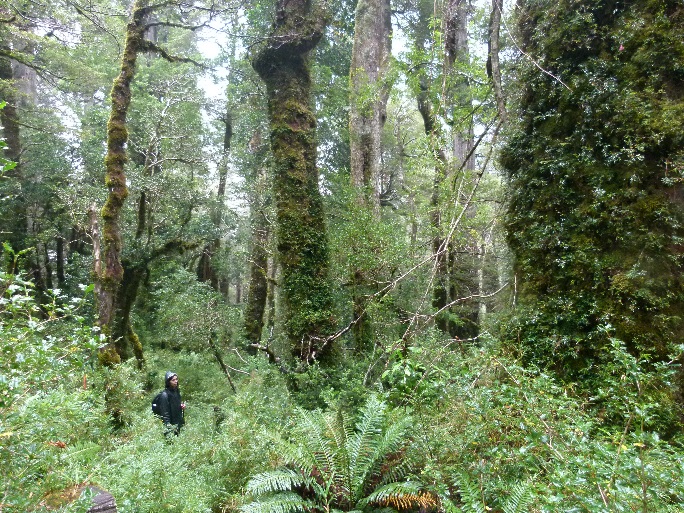 |

**Figure A.** Structure of the studied forests at top: the Alerce Costero National Park and bottom: Alerce Andino National Park (Photos: Yadvinder Malhi).

**Figure B.** Mean monthly radiation, temperature, relative humidity, soil temperature, and total monthly precipitation in Alerce Costero (AC) and Alerce Andino (AA) between November 2011 and October 2012. White dots in the figures indicate values estimated from climate data from the weather station at Puerto Montt. Error bars indicate ±1 SD.

**Figure C.** a-d) Diameter distribution of all species and *Fitzroya* trees in AC1 and AC2 plots. e-h) Basal area for the same components in both plots.

|  |  |
| --- | --- |

**Figure D.** a-d) Diameter distribution of all species and *Fitzroya* trees in AA1 and AA2 plots. e-h) Basal area for the same components in both plots.

**Figure E.** a) Total woody biomass for trees ≥2 cm DBH along different diameter classes in each plot, b) The same as a), but for total woody productivity.

**Figure F.** Aboveground coarse wood productivity (NPP_ACW_) and mortality in trees ≥ 10 cm DBH in the Coastal (a) and the Andean plots (b). FC: *Fitzroya cupressoides*, DW: *Drimys winteri*, NN: *Nothofagus nitida*, MC: *Myrceugenia chrysocarpa*, SC: *Saxegothaea conspicua*.

**Figure G.** The seasonal cycle in branchfall (every three months) in each plot between February 2012 and November 2012. The month of February 2012 includes branches fallen in the period November 2011-February 2012. Error bars show ±1 SE.

**Figure H.** The seasonal cycle in canopy litterfall (bottom) and its components (non-*Fitzroya* leaves, needles, reproductive material and twigs) between November 2011 and October 2012. Error bars show ±1 SE.

**Figure I.** Litterfall per component (in Mg C ha^-1^ year^-1^) per plot.
